# Deciphering Cancer Cell Behavior From Motility and Shape Features: Peer Prediction and Dynamic Selection to Support Cancer Diagnosis and Therapy

**DOI:** 10.3389/fonc.2020.580698

**Published:** 2020-10-20

**Authors:** Michele D'Orazio, Francesca Corsi, Arianna Mencattini, Davide Di Giuseppe, Maria Colomba Comes, Paola Casti, Joanna Filippi, Corrado Di Natale, Lina Ghibelli, Eugenio Martinelli

**Affiliations:** ^1^Department of Electronic Engineering, University of Rome “Tor Vergata”, Rome, Italy; ^2^Department of Chemical Science and Technologies, University of Rome “Tor Vergata”, Rome, Italy; ^3^Department of Biology, University of Rome “Tor Vergata”, Rome, Italy

**Keywords:** machine learning, cell motility, peer prediction, dynamic feature selection, cancer heterogeneity, metastatic cancer cell detection, drug screening

## Abstract

Cell motility varies according to intrinsic features and microenvironmental stimuli, being a signature of underlying biological phenomena. The heterogeneity in cell response, due to multilevel cell diversity especially relevant in cancer, poses a challenge in identifying the biological scenario from cell trajectories. We propose here a novel peer prediction strategy among cell trajectories, deciphering cell state (tumor vs. nontumor), tumor stage, and response to the anticancer drug etoposide, based on morphology and motility features, solving the strong heterogeneity of individual cell properties. The proposed approach first barcodes cell trajectories, then automatically selects the good ones for optimal model construction (good teacher and test sample selection), and finally extracts a collective response from the heterogeneous populations *via* cooperative learning approaches, discriminating with high accuracy prostate noncancer vs. cancer cells of high vs. low malignancy. Comparison with standard classification methods validates our approach, which therefore represents a promising tool for addressing clinically relevant issues in cancer diagnosis and therapy, e.g., detection of potentially metastatic cells and anticancer drug screening.

## Introduction

The ability of cells to coordinately move is indispensable in many biological processes, such as tissue morphogenesis and repair, cancer progression, and invasion (i.e., metastasis spreading) (1). Cell movements vary according to intrinsic features and microenvironmental conditions, possibly being a signature of underlying biological phenomena. A straightforward simplification is that, for instance, healthy cells move differently from tumor cells, especially when they undergo the epigenetic changes leading to epithelial-to-mesenchymal transition, a phenomenon that provides new motility ability to cancer cells allowing metastatic spreading (2). Motility is hardly described by mere molecular markers, and therefore this important issue requires different approaches to be properly addressed.

Classifying cells according to their behavior in terms of coordinated motility needs facing the problem of cell heterogeneity; cells apparently identical by morphological criteria may behave differently because of fundamental differences in genetic or epigenetic asset, the stage of cell cycle or differentiation, in cell–cell or cell–environment interaction, etc., parameters that, although assessable by single molecular labeling, continuously change in time and combinations, being thus impossible to describe in classical molecular terms. Heterogeneity in cell response thus represents a big limitation to identify the underlying biological scenario from cell motility; nevertheless, such heterogeneity allows extracting behavioral rules to finalize the automatic understanding, for example, of cell state (e.g., tumor vs. nontumor), tumor stage (e.g., metastatic vs. nonmetastatic), response to anticancer drugs, etc. To this purpose, label-free (3) fluorescence time-lapse microscopy (TLM) and special purpose video data analysis tools (4–7) are providing promising novel, nonmolecular, dynamic approaches.

We present here a novel methodology to conduct massive analysis of cell motility in different *in vitro*–controlled conditions that combines TLM and label-free imaging, with cell tracking, quantitative representation of trajectories, and novel machine learning (ML) strategies within peer prediction framework. Peer prediction strategies acquired much interest in many contexts such as assignments in massive open online courses and in collecting feedback about a new service (8). Such algorithms use reports from multiple participants to score their contributions in settings in which there is no way to verify the quality of response (9). Cell systems, where a unique correct response for cell behavior is not expected, represent therefore an unconventional and challenging environment for peer prediction paradigm extension.

Optimization of ML strategies and adaptation to cell motility investigation need the identification of the correct learning examples. Differently from other social contexts (10, 11), none of the cells and related trajectories can be judged by experts, both because it cannot be practically done and because the heterogeneity of cell behavior and the massive number of cells make it impossible to extract the “truth” at sight. Because the acquired samples (cells) are not labeled by experts, cell trajectories would directly inherit the same label assigned to the entire experiment, i.e., cells moving in a control experiment would be assumed to behave in a unique, similar way. However, this assumption is generally invalid. The intrinsic data heterogeneity forbids the direct assignment of a unique label to all the cells, impeding to represent a cell population as a unique behavioral entity. Hence, the selection of samples for model construction becomes the core of the ML problem.

In the present work, we address the problem of learning a classification model from cell trajectories and related descriptors (peers) using a novel strategy. First, inspired by a previous approach (12), all cell trajectories are “barcoded” during model construction; however, only some of the barcoded trajectories are assigned the role of trainers (hereafter denoted as “the good teachers”) because only a certain number of cell trajectories can be used to construct the good model. Second, not all cell trajectories in the test set are used for testing because not all of them represent the global target (e.g., a unique behavior for the same cell line or the same reaction to a given stimulus). The presence of a collective response phenomenon forces the approach to automatically identify peers in the test set, with high agreement in terms of the same descriptors selected in the training step.

Regarding the descriptors selection, only some features extracted from each cell trajectory can be assigned a “discriminatory role” because not all features are likely to be simultaneously relevant for all groups of cells. As an example, in a group of cells moving toward a target cell, e.g., immune–cancer cross-talk (13, 14), speed and directional persistence are needed to model their collective motion; on the other hand, in a group of cells interacting with a target cell, e.g., immune cells killing a cancer cell (15, 16), mean interaction time and track curvature have proved to be specifically tailored for the phenomenon quantification. In particular, in this work, we extended and applied a dynamic feature selection (DFS) procedure (17, 18), selecting, in an unsupervised way, the optimal feature set extracted from the training set for each new test sample; this will be used to build a classifier for the test label prediction.

Of importance, in addition to the model construction, in our approach the novelty includes the decision-making step. In *in vitro* experiments, cells naturally cluster before reaching the confluence; consensus strategies can be exploited to acquire a unique decision for the cluster. In this regard, we applied two distinct cooperative learning criteria, inspired by collective phenomena and peer influence studies (11); on the one hand, we applied a majority voting procedure to all the labels assigned by the classifier to the cell trajectories selected for that cluster; on the other, we summed up all the scores assigned to each category of the cells belonging to the same cluster and assigned the class with the largest total score to the cluster. We refer to the two criteria as majority voting criterion (maj-vot) and maximum trustiness criterion (max-trust).

## Materials and Methods

### Video Acquisition Details

The videos were acquired with a custom small-scale inverted microscope (19). In order to have control on acquisition methods and light exposure, a custom firmware was developed in MATLAB 2017a®. We acquired images at one frame per minute with 6 h of total experimental time (12 h in the LNCaP case). The images have a field of view of 1.2-mm width by 1.0-mm height and a theoretical spatial resolution of 0.33 μm/px.

We recorded two videos per treatment condition in RWPE-1 and PC-3 prostate cell experiments and four videos for the control case in the LNCaP cells.

### Cell Culture Details

Human prostate cancer cells, PC-3 and LNCaP cell lines (ATCC, Rockville, MD), were grown in RPMI 1640 medium, supplemented with 10% fetal bovine serum, 1% l-glutamine (2 mg/mL), and 1% penicillin/streptomycin (100 IU/mL) (Euroclone).

Nonneoplastic, immortalized human prostatic epithelial cells, RWPE-1 (ATCC, Rockville, MD) were grown in keratinocyte serum-free medium (K-SFM), supplemented with 1% penicillin/streptomycin (100 IU/mL), 50 μg/mL bovine pituitary extract, and 5 ng/mL epidermal growth factor (Life Technologies, Barcelona, Spain).

Cells were grown at 37°C in a humidified atmosphere of 5% CO_2_ in air. In each experiment 40,000 cells/mL were seeded in 35-mm Petri dishes (Jetbiofil). Seventy-two hours postseeding, cells were treated with the chemotherapeutic drug etoposide (Sigma-Aldrich), a topoisomerase II inhibitor, at the final concentrations of 0.5, 1, or 5 μM and immediately analyzed with TLM.

### Method for Automatic Cell Behavior Classification

#### Step 1. Cell Localization and Tracking

The method is focused on the use of a previously validated cell tracking tool, Cell-Hunter, which has been tested in prostate cancer cell automatic tracking (12, 19), immune–cancer cell crosstalk studies (16), and recently in red blood cell plasticity analysis (20). The software automatically locates cells with a radius within a given range provided by the user and tracks them providing a predetermined maximum displacement allowed.

#### Step 2. Automatic Cell Clustering Identification

Cells naturally cluster when they are put in *in vitro* culture, a primitive status before moving toward confluence. Cells move according to the cluster they belong, promoting different roles according to the cell stage, age, drug absorption, etc. The automatic identification of the clusters each cell belongs to is performed through image analysis algorithms involving image binarization and morphological operators (12). The technique is based on the localization of individual cells by performing the segmentation of circular objects using the Circular Hough Transform (CHT) (21) set according to the mean estimated radius of cells involved. Each detected cell is represented as a white circular object. By using an accumulation criterion, consisting of the overlapping of the cell nuclei detected along all the frames and normalizing by the maximum value, a gray-scale map is obtained, in which higher intensity values locate cells with limited motility frame by frame and thus higher probability to stay in that position during movement. By applying pixel intensity thresholding using the Otsu criterion (21) and then morphological operators refining (21), a rough binary (black and white) image representation of each cluster is obtained. The boundary extraction of the detected regions represents cluster contours.

#### Step 3. Feature Extraction

Each cell is characterized in terms of its kinematics and shape dynamics. To do this, we identified some quantitative descriptors to characterize the dynamics of cell movement. In addition, shape descriptors are also considered to characterize the morphodynamics during movement. Further mathematical details of the two sets of descriptors can be found in the following subparagraph.

##### Cell morphology feature extraction

The shape extraction process is described in Supplementary Figure 1. We used the position of the cell trajectory to correctly focus the window containing the cell under study for every frame (Supplementary Figure 1A). We obtained an initial contour applying a CHT (22, 23) with a high sensitivity and a maximum radius smaller than the radius expected from the first object found (Supplementary Figure 1B). We took the perimeter of the smallest convex polygon (convex hull) containing the union of all the found circles (Supplementary Figure 1C) as starting contour for an active contours algorithm (24) that gave us the final result (Supplementary Figure 1D).

Looking at time-lapse videos, we observed that cells in their motion change eccentricity, perimeter, and area. They also change solidity when making pseudopodia. Furthermore, nonneoplastic cells (RWPE-1) are smaller than the others, and the milder neoplastic cells (LNCaP) have a higher eccentricity on average. These considerations led us to consider as significant features eccentricity, area, perimeter, and solidity (25).

(a) Eccentricity is defined as

(1) $$\begin{array}{l} \text{eccentricity}=\frac{d_{f}}{D_{M}} \end{array}$$

where *d*_*f*_ is the distance between the foci, and *D*_*M*_ is the major axis length;

(b) Area is defined as

(2) $$\begin{array}{l} \text{area}=\underset{i}{\sum }\underset{j}{\sum }f\left(i,j\right) \end{array}$$

where *f*(*i, j*) is 1 for (*i, j*) in the region of interest and zero elsewhere;

(c) Perimeter

(3) $$\begin{array}{l} \text{perimeter}=\underset{i}{\sum }\underset{j}{\sum }g\left(i,j\right) \end{array}$$

where *g*(*i, j*) is 1 over the pixels that have at least one neighbor (in 8-connection) with zero value and zero elsewhere;

(d) Solidity (26) is defined as

(4) $$\begin{array}{l} \text{solidity}=\frac{\text{area}}{\text{area convex hull}} \end{array}$$

where the convex hull is the smallest convex polygon that contain the region.

To exploit the dynamic of these descriptors for each cell over time, we performed the following statistics: mean, standard deviation, skewness, kurtosis, Shannon entropy, and signal entropy.

##### Cell motility feature extraction

In order to have statistical significance of the extracted features, we discarded all the trajectories, which lasted <50 min (50 time points). Cell position at each time point is affected by errors: discretization error, which is linked to the dimension of each pixel (0.66 μm/px) and the optical resolution (*R* ≅ 0.8 μ*m*). Another source of error occurs when the algorithm does not find the cell, assigning the previous position to the cell, thus resulting in jumps in the trajectories. We reduced this error with a smoothing spline approximation (27). On the new set of coordinates, (*x*_*s*_(*t*_*k*_), *y*_*s*_(*t*_*k*_)), we computed the following parameters for their already proven informative content (19):

(i) Tangential speed norm

(5) $$\begin{array}{l} v\left(t_{k}\right)=\sqrt[{\;}]{{\left(\frac{x_{s}\left(t_{k+1}\right)-x_{s}\left(t_{k}\right)}{\left(t_{k+1}-t_{k}\right)}\right)}^{2}+{\left(\frac{y_{s}\left(t_{k+1}\right)-y_{s}\left(t_{k}\right)}{\left(t_{k+1}-t_{k}\right)}\right)}^{2}\;} \end{array}$$

(ii) Track curvature χ (*t*_*k*_)

(6) $$\begin{array}{l} \chi \left(t_{k}\right)=\frac{\left|{x_{s}}^{\prime }{y_{s}}^{\prime \prime }-{y_{s}}^{\prime }{x_{s}}^{\prime \prime }\right|}{{\left[{\left({x_{s}}^{\prime }\right)}^{2}+{\left({y_{s}}^{\prime }\right)}^{2}\right]}^{\frac{3}{2}}} \end{array}$$

(iii) Turning angle ϑ (*t*_*k*_)

(7) $$\begin{array}{l} \vartheta \left(t_{k}\right)=\tan^{-1}\left(\frac{v_{x}}{v_{y}}\right) \end{array}$$

(iv) Angular velocity, computed as the ratio between the magnitude of the velocity and the distance from the center of the trajectory.

(8) $$\begin{array}{l} \omega \left(t_{k}\right)=\frac{v\left(t_{k}\right)}{R\left(t_{k}\right)} \end{array}$$

where $R\left(t_{k}\right)=\sqrt[{2}]{{\left(x_{s}\left(t_{k}\right)-x_{c}\right)}^{2}+{\left(y_{s}\left(t_{k}\right)-y_{c}\right)}^{2}}$, $x_{c}\;=\frac{1}{N}\overset{N}{\underset{k=1}{\sum }}x_{s}\left(t_{k}\right)$ and $y_{c}=\frac{1}{N}\overset{N}{\underset{k=1}{\sum }}y_{s}\left(t_{k}\;\right)$.

(v) Diffusion coefficient

(9) $$\begin{array}{l} D=4^{-1}\cdot e^{y_{0}} \end{array}$$

where *y*_0_ is the *y*-axis intercept estimated form a linear fit in log space of the mean square displacement (28).

(vi) Directional persistence, defined as the ratio between the initial and the final point and the real length of the track.

(10) $$\begin{array}{l} p=\frac{\left\|{\underline{x}}_{s}(t_{f})-{\underline{x}}_{s}(t_{i})\right\|}{L} \end{array}$$

where $L=\sum_{k}\left\|{\underline{x}}_{s}(t_{k})-{\underline{x}}_{s}(t_{k-1})\right\|$.

From each time-varying feature, we extracted the following high-level statistical descriptors: mean, standard deviation, skewness, kurtosis, and signal Shannon entropy. In conclusion, we collected 24 shape descriptors and 39 motility features.

Kinematics and shape features allow excluding some trajectories from the whole analysis through unsupervised outlier detection. Such step is required because of some false tracks extracted by the cell tracking software. Misdetected trajectories may be related to false cells localization (for example, out-of-focus cells) or to tracks that exit the field of view and are linked to new cells entering the scene.

It is straightforward to note that optimal descriptors depend not only on the task, but also on the training and testing samples. For this reason, we selected a wide set of descriptors commonly used for evaluating cell behavior from motility and shape analysis. The assumption underlying the selection needs to be able to monitor different aspects of cell motility, such as speed, curvature, turning angle, persistence, etc., as well as synthetic descriptors of shape dynamics along time.

#### Step 4. Good Teacher Selection

Let us consider a set of training samples, $F=\left|\begin{array}{c} f_{1}\\ \vdots \\ f_{T} \end{array}\right|$, with *T* as the number of training samples, and ${f^{j}}_{k}=\left\{f_{k1}^{j},f_{k2}^{j},\ldots ,f_{kM}^{j}\right\}\;$the subarray of descriptors for the *k*th cell in the *j*th cluster ***C***_***j***_, *j* = 1, …*N*_*C*_ with *N*_*C*_ being the number of clusters in the training set.

First, the algorithm automatically selects a subset of descriptors, $\tilde{F}\;\subset F,\;$with *T* rows and *M*′ < *M* columns (descriptors) such that a maximum value is obtained for a given criterion Ψ_1_ applied to the set $\tilde{F}$. The suboptimal criterion Ψ_1_ used here is the maximum area under the curve (AUC) values (29) obtained in all the associated binary problems in a multiclass context (in an all-vs.-all classification strategy validated on the training set). The AUC is a metric of separability for a given descriptor with respect to the output label of different classes. The higher the AUC value (bounded in [0,1]), the higher the discrimination capability of the descriptor.

Then, a subset of training samples, namely, ***F***′ $\subset \tilde{F}$ with *T*′ < *T* rows and *M*′ columns, is extracted by taking the training samples whose descriptors fall within a tuned range (i.e., percentile [*th*_1_, *th*_2_]) independently calculated in each video. Formally, [*th*_1_, *th*_2_] allows keeping all the observations whose cumulative distribution function is between *th*_1_ and *th*_2_.

The threshold values set in each experiment are listed in Table 1, rows 1 and 2.

**Table 1:** List of algorithm parameters setting used in the experiments for performance assessment.

**Algorithm parameters setting**			
**Symbol**	**Description**	**Shape features**	**Motility features**
*th_1_*	Lower bound percentile good teacher selection	0.2	0.2
*th_2_*	Upper bound percentile good teacher selection	0.8	0.8
*th_3_*	Lower bound percentile test sample selection	0.1	0.1
*th_4_*	Upper bound percentile test sample selection	0.9	0.9
*th_1*dfs*_*	DFS Fisher criterion threshold	1.0	0.9
*th_2*dfs*_*	DFS minimum Mahalanobis criterion threshold	0.1	0.1
*th_3*dfs*_*	DFS maximum probability criterion threshold	1.0	0.2
*p*	Confidence value for stepwise feature selection	0.3	0.3

*A brief description of each parameter is also included*.

#### Step 5. Test Sample Selection

By using the same descriptors selected in Step 4, a similar refining procedure is applied to the test cell trajectories, by using an independent range of elimination, namely [*th*_3_, *th*_4_], leading to test samples indicated with ***H***. Table 1, rows 3 and 4, lists the values for percentiles *th*_3_ and *th*_4_. Good teacher and test sample selection procedures represent the forerunner of the peer prediction paradigm.

#### Step 6. Dynamic Feature Selection

**Figure 1:**
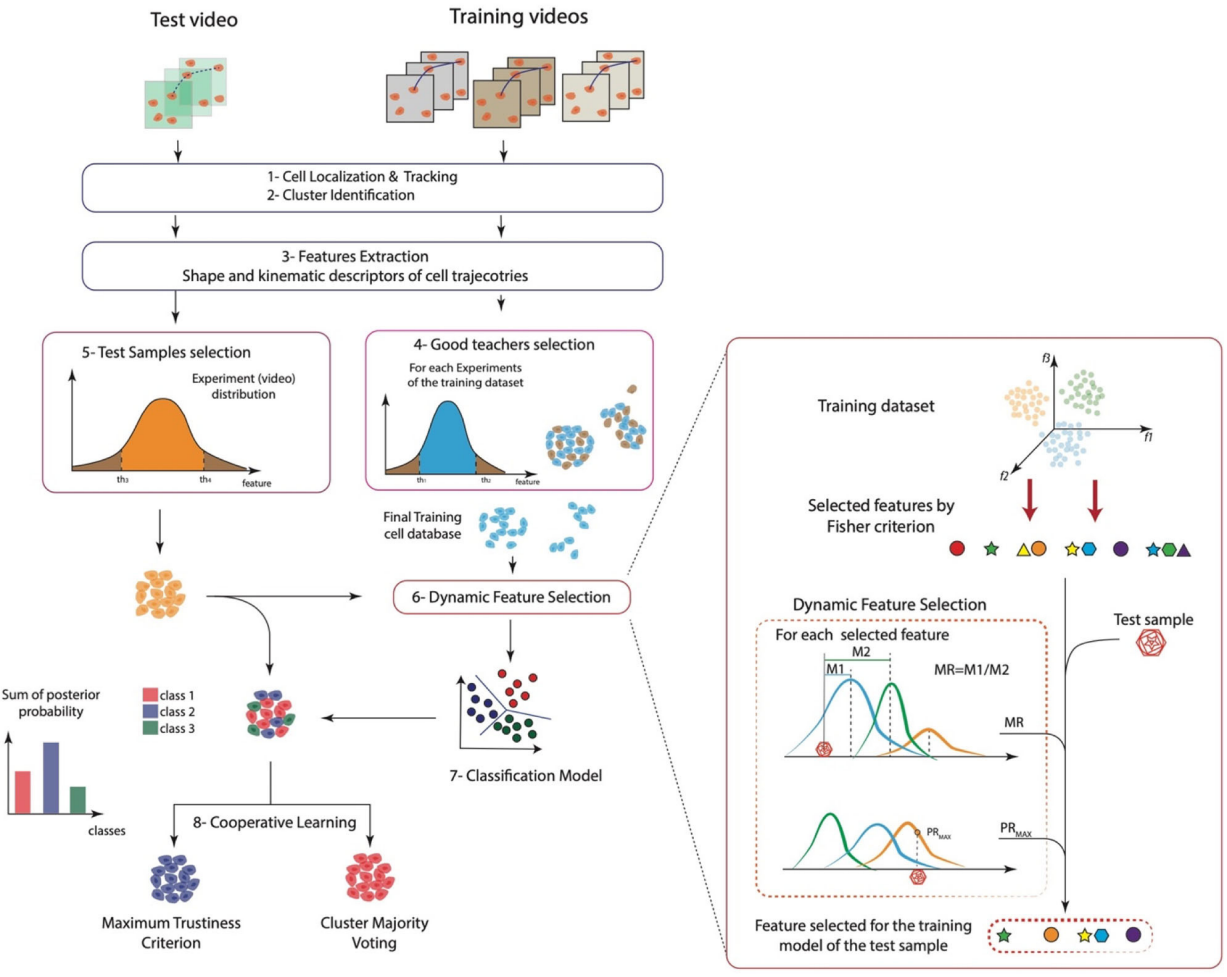
A sketch of the whole approach. (1) Cell localization and tracking, (2) cluster identification, (3) features extraction, (4) good teachers selection, (5) test samples selection, (6) dynamic feature selection, (7) classification model, and (8) cooperative learning.

After training and test data have been collected, namely, ***G*** and ***H***, descriptors are finely selected by using a DFS procedure. DFS applies three distinct criteria. The first supervised criterion, Fisher criterion in Figure 1, selects features that correlate with the output in training set, according to a limit value *th*_1dfs_. The second and third criteria are unsupervised and use two distinct approaches. In the second, the Mahalanobis criterion (Figure 1), features in the test set whose Mahalanobis distance from features in the training set is under a given limit threshold *th*_2dfs_ are selected. In the third criterion (Figure 1), the maximum posterior probability of feature values in test to belong to the distribution of values in training set over all the classes is calculated; features with probability values higher than a given threshold *th*_3dfs_ are kept. Further mathematical details can be found in Mosciano et al. (18). Features that satisfied all the three criteria are then selected. The extension we propose here with respect to the standard DFS (18, 30) is the inclusion of a preliminary supervised selection performed at the beginning of the model construction based on stepwise feature selection procedure (31) applied on the training samples. The fact that motion models may vary within the same experiment (12) implies the necessity to extract many kinematics descriptors. The modification to standard DFS allows limiting the initial set of descriptors to a maximum effective set. The *p*-value of the *F* test (32) used for the acceptation of a feature in the selection process, indicated with *p*, is an algorithm parameter. Values of *th*_1dfs_, *th*_2dfs_, *th*_3dfs_, and *p* are listed in Table 1, rows 5–8.

Finally, we may indicate with $\bar{G}$ and $\bar{H}$ the refined sets for model construction and automatic classification.

#### Step 7. Classification Model

Model construction is performed considering three distinct classification models: linear discriminant analysis (LDA) (33), support vector machine (SVM) (34), and K-nearest neighbor (KNN) (35).

LDA finds a linear combination of features (input data) to separate two or more classes of objects or events. In this work, LDA naturally produces as an outcome not only the class label but also an associated posterior probability to belong to the class. According to this, given a test set $\bar{H}$, the LDA model provides for each class $c_{\bar{H}}$ a score value $y_{\bar{H}}$. Such values are used in the cooperative strategies as shown below.

SVM presents one of the most robust prediction methods, based on the statistical learning framework. An SVM model is a representation of the examples as points in new prediction space, mapped so that the examples of the separate categories are divided by a clear gap that is as wide as possible. New examples are then mapped into that same space and predicted to belong to a category based on the side of the gap on which they fall. The SVM algorithm may be turned into nonlinear classification model by using a nonlinear kernel, commonly radial basis function. In this work, we used SVM with linear kernel for harmonization with the LDA competitive method.

KNN is a nonparametric method in which the input consists of the K-closest training examples (*K* = 5 in this work) in the feature space (input data), whereas the output is a class membership. An object is assigned to the class most common among that of its KNN training samples. A standard metric for representing neighborhood is the Euclidean distance, which is used in our work.

#### Step 8. Cooperative Learning

In the test set, all the cell trajectories associated to a cluster are individually scored through $y_{\bar{H}}$ and labeled through $c_{\bar{H}}$. Under the need to provide a unique decision, i.e., a unique proof of concept to the underlying biological hypothesis, the approach allows aggregating the labels and the scores of the trajectories belonging to the same cluster, using cooperative decision-making strategies. In details, we considered two distinct independent criteria that are used as alternatives. On the one hand, counting of labels $c_{\bar{H}}$ assigned to each class in the cluster is applied, and the class with the majority of labels is finally assigned to the cluster, the *majority voting criterion*. On the other hand, the sum of scores $y_{\bar{H}}$ assigned to each class computed over the cluster is used to assign the class with the highest score, the *maximum trustiness criterion*.

The two criteria are inspired by two different considerations. First, *maj-vot* represents the logic of consensus based on the agreement among artificial labelers (cell trajectories in test). This is in line with the assumption of a unique collective underlying phenomenon in a given experiment. On the other hand, the *max-trust* criterion considers all the scores assigned to the entire cluster giving strength not only to artificial labelers in agreement (majority voting paradigm) but rather to all labelers in the cluster, even those not in agreement. In other words, the latter criterion applied a more democratic principle, giving voice also to minority cell behavior with high scores. Cooperative learning approaches represent the final step of the peer prediction paradigm, in which final decision is taken among peers, after the elimination of abnormal or deviated responders (test samples rejected).

### Experimental Setup

Three prostate cell lines were chosen to test the validity of the proposed methodology: RWPE-1 (nonneoplastic cells), LNCaP (neoplastic cells), and PC3 (metastatic neoplastic cells), representing healthy, tumor, and highly aggressive tumor cell phenotypes, respectively. RWPE-1 and PC3 were treated with the chemotherapy agent etoposide at different concentrations (0.5 and 5 μM for RWPE-1, 1 and 5 μM for PC3). RWPE-1 and PC3 were also acquired in control conditions (i.e., no drug). Therefore, for RWPE-1 and PC3, we collected six videos (two ones for each condition), and for LNCaP, we collected four replicated experiments in control condition (globally 16 videos).

In order to demonstrate the effectiveness and the general validity of the approach, we ran a leave-one-experiment-out validation procedure, holding out an experiment at a time for testing and using the remaining for training the method. Despite the low number of available experiments, results are very promising, in relation to the challenging identified setup. On the other hand, under the assumption of the intrinsic heterogeneity of the cell behavior in a given group of nominally identical cells, we performed cooperative learning by maj-vot and max-trust criteria applied at cluster level.

An example of clustered cells for the three cell lines is shown in Supplementary Figure 2. The color bar indicates the time varying cross the trajectory. Four distinct cell shapes and positions along the corresponding trajectories are also shown. As immediately observed, cell appearance is very heterogeneous, both among the same cell line and along the trajectory of the same cell. This fact demonstrates the difficulty to extract synthetic descriptors from trajectories and construct a model on them for recognizing changes in the cell behavior.

### Quantification and Statistical Analysis

To evaluate the performances of all the classification models, a cross validation procedure has been applied.

## Results

### Setting of the Proposed Approach

In this work, we present a general method to analyze and discriminate cell behavior in controlled *in vitro*–cultured environments. The proposed approach can be divided into eight key steps: (1) cell localization and tracking, (2) automatic cell clustering identification, (3) cell morphology and motility feature extraction, (4) good teacher selection, (5) test samples selection, (6) DFS, (7) classification model, and finally (8) cooperative learning. A schematic representation of the whole approach is reported in Figure 1. Briefly, the method exploited a previously validated cell tracking tool (Cell-Hunter) to automatically locate and track cells. Each cell is then identified as a member of a cell cluster by image analysis algorithms (12) and characterized in terms of kinematics and shape descriptors. To this aim, quantitative descriptors to characterize cell morphology and motility over time were extracted. Good teacher and test sample selection procedures were then applied to retain only those cell trajectories considered as good trainers and good samples, respectively, to construct the model. After training and data collection, DFS further finely selected only those features satisfying the Fisher criterion, Mahalanobis criterion, and the maximum posterior probability, excluding all abnormal behaviors. Model construction was then performed, and two cooperative learning techniques, i.e., the maj-vot and the max-trust, were implemented to ultimately extract a unique collective cell response. Further details on the proposed method for automatic cell behavior classification are reported in Materials and Methods.

**Figure 2:**
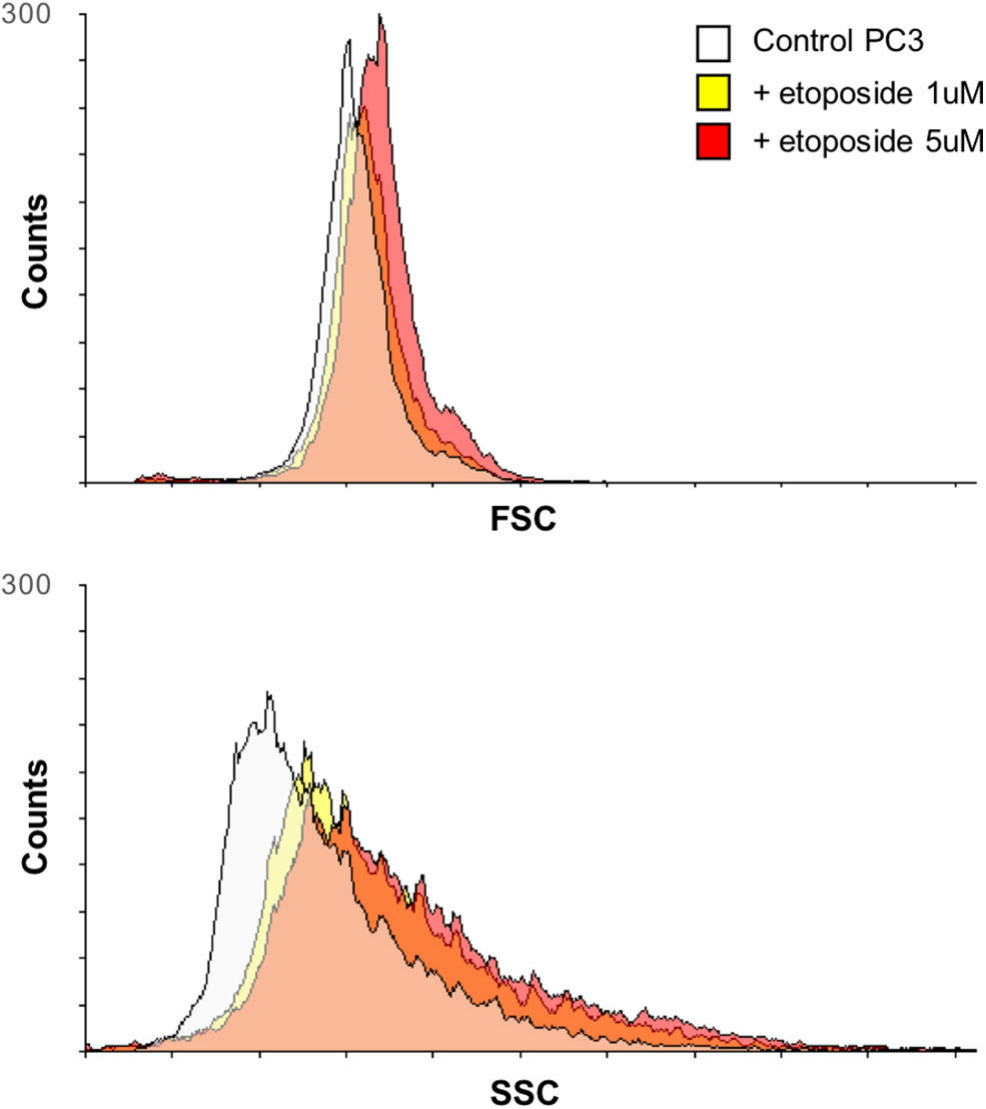
Variation over time of PC3 cell morphology after treatment with etoposide assessed by flow cytometry. Overlays of the forward scatter (FSC) and the side scatter (SSC) of PC3 cells, before and after treatment with etoposide (1 or 5 μM) for 12 h, are reported; the two parameters relate to cell size and granularity, respectively.

Three prostate cell lines were chosen to test the validity of the proposed methodology: RWPE-1 (nonneoplastic cells), LNCaP (neoplastic cells), and PC3 (metastatic neoplastic cells), representing healthy, tumor, and highly aggressive tumor cell phenotypes, respectively, treated with increasing doses of the chemotherapy agent etoposide. Among chemotherapeutics, etoposide was selected because of its well-known effect on both cell shape (Figure 2) and motility (19); i.e., it affects the features extracted for the classification method.

### Setting of Algorithm Parameters

Quantitative results of the test have been assessed using different indices; balanced accuracy and unbalanced accuracy (ACC_b_ and ACC, respectively) were computed over the confusion matrix related to the classification results. We reported the results computed over each single-cell trajectory tested (single-cell result) and the results achieved using the cooperative learning strategies. In particular, we show results referred to the maj-vot and to the max-trust criteria. Furthermore, the results were compared with those obtained using standard classification strategies or with the elimination of specific algorithms blocks, such as data test and good teacher (training and/or test) selection. In this way, we demonstrated the validity not only of the whole approach, but also the improvement introduced by each sub-block.

Table 1 lists the parameters values for each test performed for system performance assessment. The values have been estimated by an optimization procedure run on a repeated subsampling of the training set.

### Classification Results: The Proposed Method Reached Accuracy Values of 95%

We validated the approach on the automatic recognition of the three different prostate cell lines tested (RWPE-1, LNCaP, and PC3). We separated the results obtained using only shape or motility features, in order to appreciate the relevance of the two groups of descriptors for the task.

**Figure 3:**
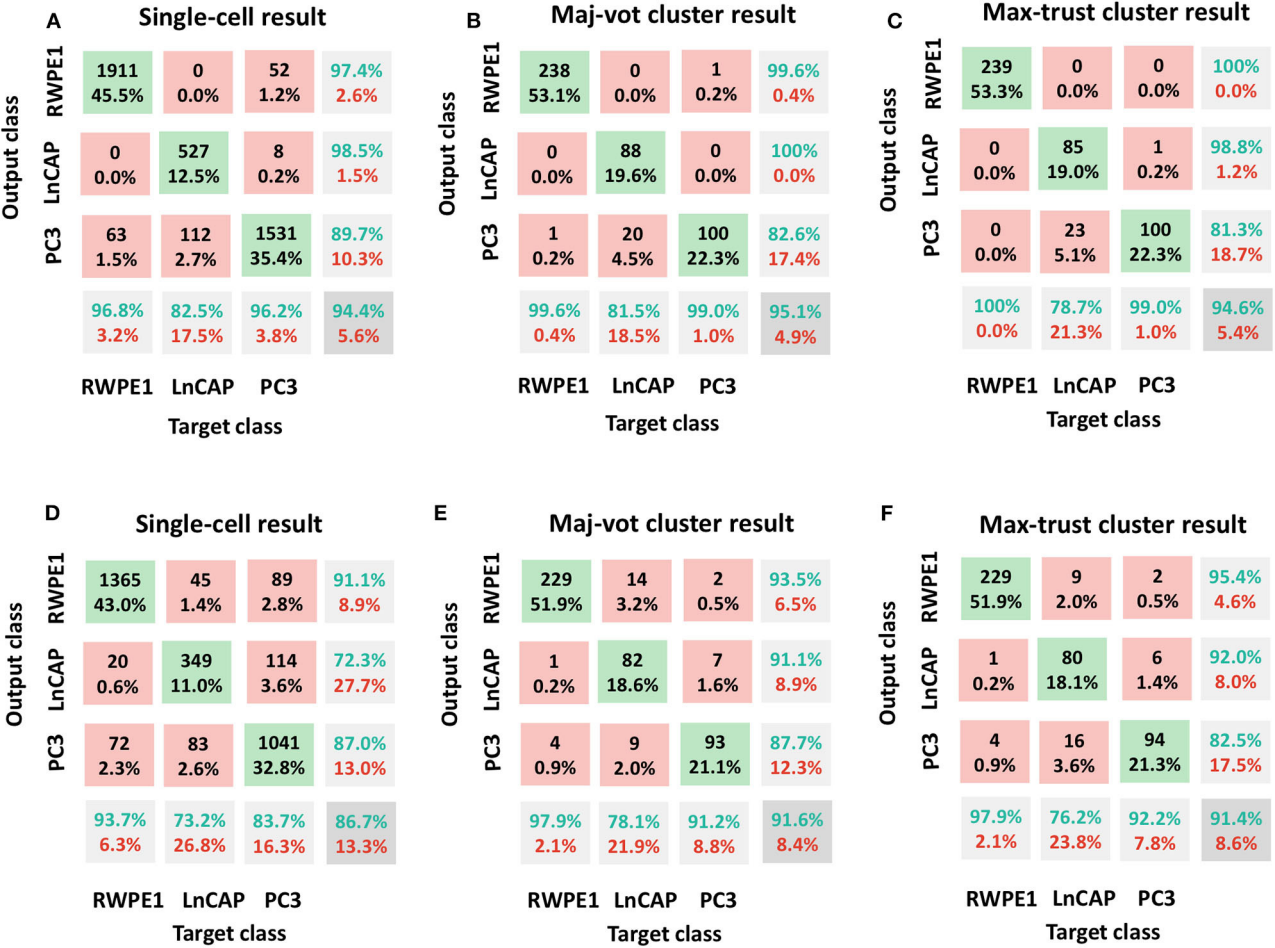
Classification results: RWPE1, LNCaP, and PC3 cell lines. Shape features in **(A–C)** and motility features **(D–F)**. **(A,D)** Single-cell result, **(B,E)** maj-vot cluster-level result, **(C,F)** max-trust cluster-level result. In **(A,D)**, numbers indicate the cells tested, whereas in **(B,C,E,F)**, numbers indicate the number of clusters. Green accuracy values represent the true-positive results for each class, whereas the values in the pink boxes indicate the number of false positives (upper triangular part of the confusion matrix) and false negatives (the lower triangular part of the matrix). The values in the gray box represent the total accuracy.

In Figure 3, we included the confusion matrices using the SVM classifier related to (A, D) single-cell result, (B, E) maj-vot result performed at cluster level, and (C, F) max-trust result performed at cluster level, for shape (A–C) and motility (D–F) features, respectively.

In detail, by using shape descriptors, we obtained accuracy values, ACC (ACC_b_), equal to 94.4% (91.8%) for the single-cell result, 95.1% (93.4%) for the maj-vot result, and 94.6% (92.6%) for max-trust result. The highest accuracy values are obtained for RWPE-1 and LNCaP cells. PC3 cells, instead, are misclassified in more than 10% of cases. Nevertheless, the classification error always occurs in the LNCaP class and never in that of the RWPE-1, underlying the great validity of the novel approach that when it fails, it misclassifies only between the two tumor classes (metastatic vs. nonmetastatic tumor cells), in accordance with the heterogeneity-characterizing tumors.

By using only motility features, instead, we obtained lower (although still very promising) accuracy values, ACC (ACC_b_), equal to 86.7% (83.5%) for the single-cell result, 91.6% (89.0%) for the maj-vot result, and 91.4% (88.8%) for max-trust result.

The use of shape descriptors therefore improves the global recognition accuracy with respect to motility features. This is a further demonstration of the potential of video analysis in TLM toward the possibility to combine spatiotemporal properties in morphokinetic studies.

### The Crucial Role of the Good Teacher and Test Sample Selection to Maximize the Classification Performance

In this section, we evaluated the results of the proposed approach based on the three distinct classification models: LDA, SVM, and KNN.

Classification results are shown in Table 2. As it can be noted, the three classifiers produced similar results (above all LDA and SVM); cell classification according to the phenotype is effectively solved by the proposed synergic approach. In light of this, LDA remains the simplest model achieving almost the highest performance, to the advantage of an increased architecture and easier interpretation of the results.

**Table 2:** Comparative results in terms of balanced accuracy (ACC_b_) and accuracy (ACC) of classification.

		**(A)**	**(B)**	**(C)**	**(D)**	**(E)**
**Shape features**		**Proposed approach**	**SVM**	**KNN**	**No good teacher selection**	**No data test selection**
ACC_b_	Single-cell	91.94%	92.19%	88.51%	77.19%	85.15%
ACC		94.31%	93.63%	90.10%	79.84%	87.43%
ACC_b_	Maj-vote	93.36%	93.15%	92.53%	84.29%	89.95%
ACC		95.09%	95.09%	94.64%	87.53%	91.40%
ACC_b_	Max-trust	93.52%	92.55%	92.57%	86.02%	90.26%
ACC		95.31%	94.64%	94.64%	88.36%	91.61%
**Motility features**		**Proposed approach**	**SVM**	**KNN**	**No good teacher selection**	**No data test selection**
ACC_b_	Single-cell	83.51%	85.84%	80.20%	70.15%	69.70%
ACC		86.69%	88.74%	86.06%	74.62%	74.30%
ACC_b_	Maj-vote	89.04%	90.54%	86.21%	77.91%	76.56%
ACC		91.61%	92.52%	89.57%	83.43%	81.21%
ACC_b_	Max-trust	88.74%	90.10%	88.47%	80.61%	80.20%
ACC		91.38%	92.06%	91.16%	85.25%	83.64%

*Top part shows shape features, and bottom part shows motility features. The results of the proposed approach using LDA (A) are compared with the use of (B) support vector machine (SVM) with linear kernel, (C) K-nearest neighbor (KNN) with K = 5, and the cases (D) with no data selection and (E) that in which the good data selection was not applied to the test set*.

In order to demonstrate the crucial role of the good teacher and test sample selection, we conducted two specific tests. First, we totally removed the good teacher selection procedure (Step 4) from the strategies and reported the results of a model constructed on the entire training dataset and the test conducted on all the samples in each cluster. Second, we removed the test sample selection (Step 5); namely, we only selected good trainers but not good samples for testing the results. Numerical results are shown in Table 2, columns D and E.

First, we observed that using shape descriptors, performance is higher. This is due to the fact that although cell shape changes during movement, as observed from Supplementary Figure 2, and that etoposide administration deeply affects cell shape, this variation is smaller than that existing among distinct cell lines. Therefore, the impact of data selection is strong, but not crucial (we obtained even accuracy values of 88 and 92% without the application of the novel strategies). Data selection, instead, acquires a primary role in the case of motility descriptors; indeed, it increases the accuracy values even by more than 10%.

**Figure 4:**
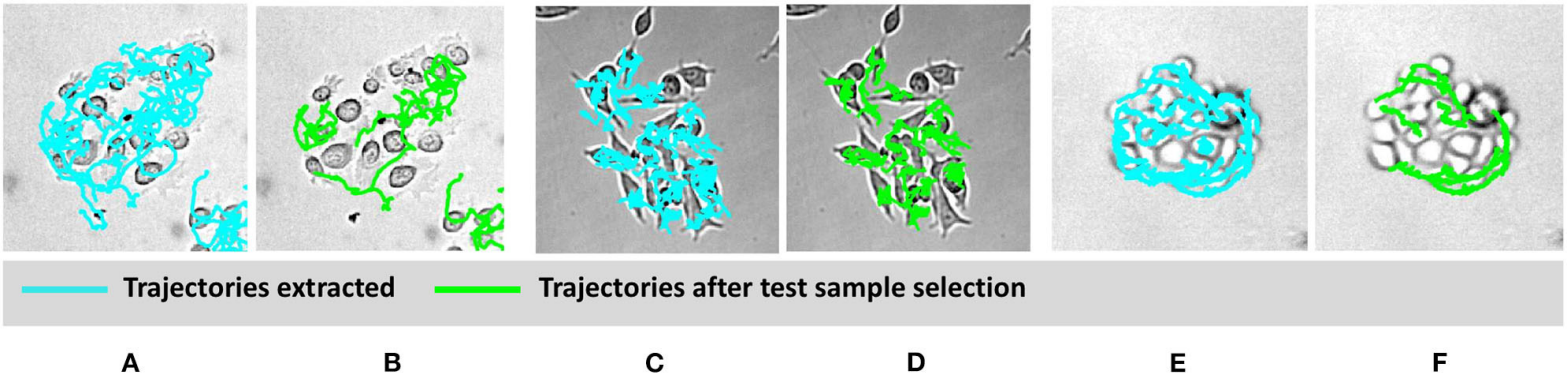
Visual example of selected cell trajectories. RWPE1 **(A,B)**, LNCaP **(C,D)**, PC3 at drug concentration of 1 μM **(E,F)**. The cyan trajectories are those extracted by the cell-tracking software for all the cells in the experiment. The green trajectories are those selected in the good sample selection.

To classify cell types based on motility features, selection of appropriate cell trajectories results pivotal; indeed, some aspects of cell behavior can be relevant for identifying a certain phenomenon, but less important for a different task. In light of this, Figure 4 shows some examples of clusters and related trajectories for the three cell lines. Using different colors for cell candidates, we could discriminate among cell trajectories extracted through the Cell-Hunter software (cyan) and tracks extracted using the test sample selection approach (green). As can be observed, in most cases, cell trajectories selected for the scope of classification as good test samples lie at the boundary of the cluster (this is particularly evident for RWPE-1 and PC3 cells), suggesting that the behavior of cells within the cluster has a less discriminative role in this case study.

## Discussion

In this work, we present a novel methodology combining TLM with cell tracking, providing a quantitative representation of trajectories and novel ML strategies, within peer prediction paradigm. This allows classifying cells in the categories of nontumor, tumor with no metastatic power, and tumor with high metastatic power, on the basis of cell behavior in terms of variations over time of cell morphology and motility.

As any methodology based on ML, we had to consider that such investigations need the identification of the correct learning examples (8). This is a hard task because of the dramatic heterogeneity of cell response, even among apparently similar cells, because of intrinsic different genetic and/or epigenetic assets and extrinsic environmental conditionings. Peer prediction protocol was implemented here to solve the strong heterogeneity of individual cell properties and activity, which renders difficult to represent a cell population as a unique behavioral entity.

To this purpose, during model construction, good teacher selection (12) was applied to cell trajectories; i.e., only those cell trajectories considered as good trainers were selected to construct the good model. The good teacher selection strategy acts therefore as a sort of candidate selection and can be used to visually investigate the role of each selected cell within any cell cluster. Selection was performed again in the testing phase: the test sample selection, indeed, allows excluding cell trajectories not complying with the representative behavior of the examined population, excluding the “noncanonical” behaviors to maximize the classification performances. Importantly, this methodology paves the way to future studies including those cells that behave differently, which could, nonetheless, represent second, third, etc., subpopulations in a heterogeneous mixture. The analysis of the currently labeled but excluded peers, in fact, would be crucial, for example, to investigate the heterogeneous genetic and epigenetic nature of cells within real biological systems, distinguishing between subpopulations. This is especially important in tumors, known to be composed of different cancer cell subpopulations. This is a paramount issue, because cancer cell heterogeneity is a main reason why therapies fail. Importantly, there are presently no straightforward ways to point out diversity. Therefore, the development and validation of the present tool, providing a mean to “barcoding” the different cancer populations, would find immediate application in clinics, with important diagnostic improvements.

To build the classifier for the test label prediction, we then combined the good teacher–good test sample selection strategies to a novel use of the DFS approach (17, 18); extracted features are dynamically selected according to the testing set characteristics. This is allowed by the novel paradigm of autonomy, in which good test samples suggest the optimal descriptors to teachers for optimal working. In line with a social peer prediction paradigm, it is the responders, and not the masters of service, who decide which aspects to judge in service quality assessment.

Through the combination of a novel good teacher–good test sample selection strategies and dynamic features selection approach for optimal model construction, we were thus able to automatically select cell trajectories for both learning and testing, by excluding cells with noncanonical behavior. The implementation of two cooperative learning techniques based on distinct peer agreement rules ultimately demonstrated the existence of a collective response rather than a collection of individual responses, finally allowing our classifier to get accuracy values of even 95% for shape descriptors.

In this regard, the use of shape together with kinematic descriptors represents a further novelty of the proposed approach. Investigation of cell morphology lost importance over time because of its impossibility of being quantifiable, therefore being not objective and not objectified. In the present work, instead, we demonstrated that the use of shape descriptors improves the global recognition accuracy of the model with respect to only motility features, thus combining spatiotemporal properties in morphokinetics studies.

The promising results achieved strongly suggest that after implementation, for example, extending the study on a larger sample of tumor cell lines, the proposed model could represent a novel tool in understanding cancer, thereby facilitating diagnosis and therapy. Indeed, the proposed predictive system may be employed in diagnostics as a fast method to identify cancer cells possessing a potential metastatic behavior and classify the type, stage, and aggressiveness of a tumor, in addition to the traditional diagnostic biomarkers screened after biopsy. To this purpose, several chemotherapeutics may be rapidly tested on patients' tumor cells, to gain information from the therapy-promoted behavioral changes; this may allow classifying patients' cells according to their aggressiveness, i.e., identifying cells metastatic potential. Noteworthy, our approach accurately correlating cell physical aspects (such as morphology and motility) to cell phenotypes may also be employed to associate different cell motilities to corresponding diverse cancer driver mutations, thus not only predicting cancer cell predisposition to therapies, but also inferring information on oncogenes and/or tumor suppressors role in cancer genesis and progression.

As far as therapy is concerned, instead, the predictive model may be used as an innovative drug screening platform, to identify effective anticancer biomodulating agents (36). Indeed, sets of chemotherapeutics may be tested on aggressive tumor cells, allowing selecting those able to remodulate cell behavior, e.g., shifting cancer cells in a less malignant or even in the nontumor category (phenotypic reversion). The proposed model would therefore allow identifying those drugs able to matter-of-factually “normalize” cancer cell behavior, even allowing case-by-case analyses for personalized therapy.

## Data Availability Statement

The raw data supporting the conclusions of this article will be made available by the authors, without undue reservation.

## Author Contributions

EM, MD'O, LG, and FC designed the experiments. FC prepared and characterized the biological samples. AM, MD'O, MC, PC, CD, and EM performed the data analysis. MD'O, JF, and DD collected the experimental videos. MD'O, AM, FC, LG, and EM wrote the manuscript. All authors contributed to the article and approved the submitted version.

## Conflict of Interest

The authors declare that the research was conducted in the absence of any commercial or financial relationships that could be construed as a potential conflict of interest.
