# Supplementary material for: Deciphering Cancer Cell Behavior From Motility and Shape Features: Peer Prediction and Dynamic Selection to Support Cancer Diagnosis and Therapy
Source: Front Oncol. 2020 Oct 20;10:580698. doi: 10.3389/fonc.2020.580698 (PMC7606946; doi:10.3389/fonc.2020.580698)
Supplement: Supplementary file 1 [file Data_Sheet_1.docx]

Supplementary Material

## Supplementary Figures:

**
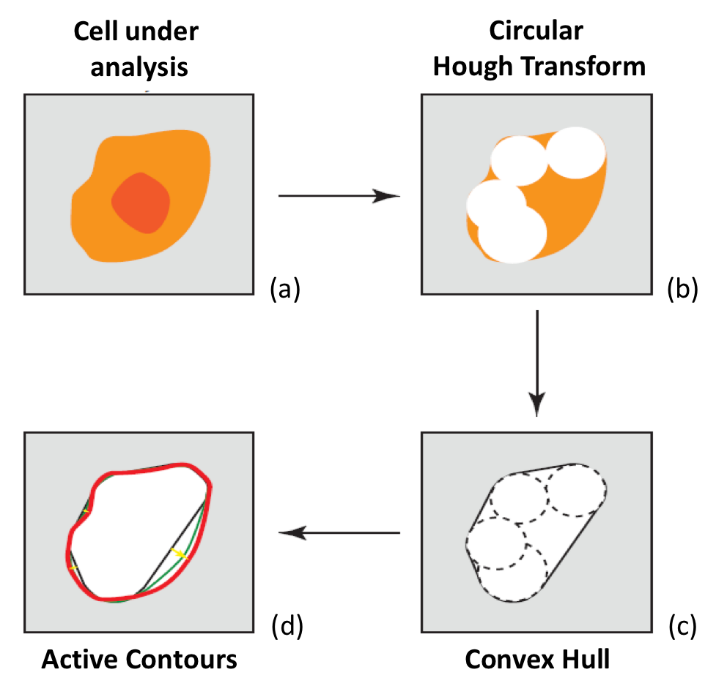
**

**Supplementary Figure 1. Flowchart of the shape extraction.** From the window containing the cell (**A**), many circles are detected using Circular Hough Transform (**B**) and a Convex Hull is Extracted (**C**). Active Contours Algorithm refines the first shape approximation obtained by the previous step (**D**).

**
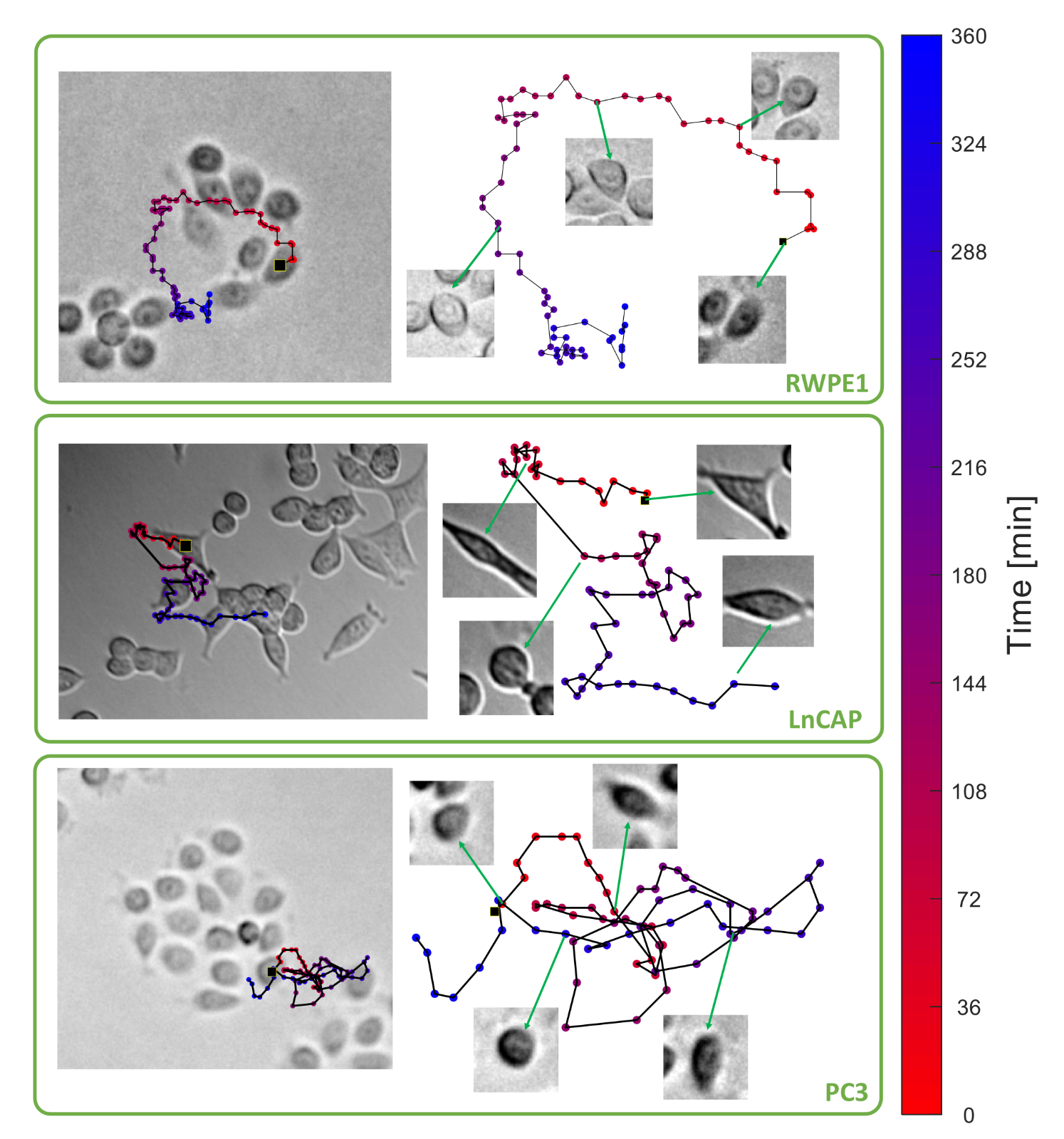
**

**Supplementary Figure 2. Visual examples of the three cell lines clusters.** Top RWPE-1, middle LNCaP, and bottom PC3 cells. Four images of the same cell along the trajectory are also indicated. The colorbar indicates temporal scale along the trajectory.
